# Supplementary material for: Contrasting Inflammatory Signatures in Peripheral Blood and Bronchoalveolar Cells Reveal Compartment-Specific Effects of HIV Infection
Source: Front Immunol. 2020 May 19;11:864. doi: 10.3389/fimmu.2020.00864 (PMC7248324; doi:10.3389/fimmu.2020.00864)
Supplement: Supplementary file 1 [file Data_Sheet_1.pdf]

Supplementary material

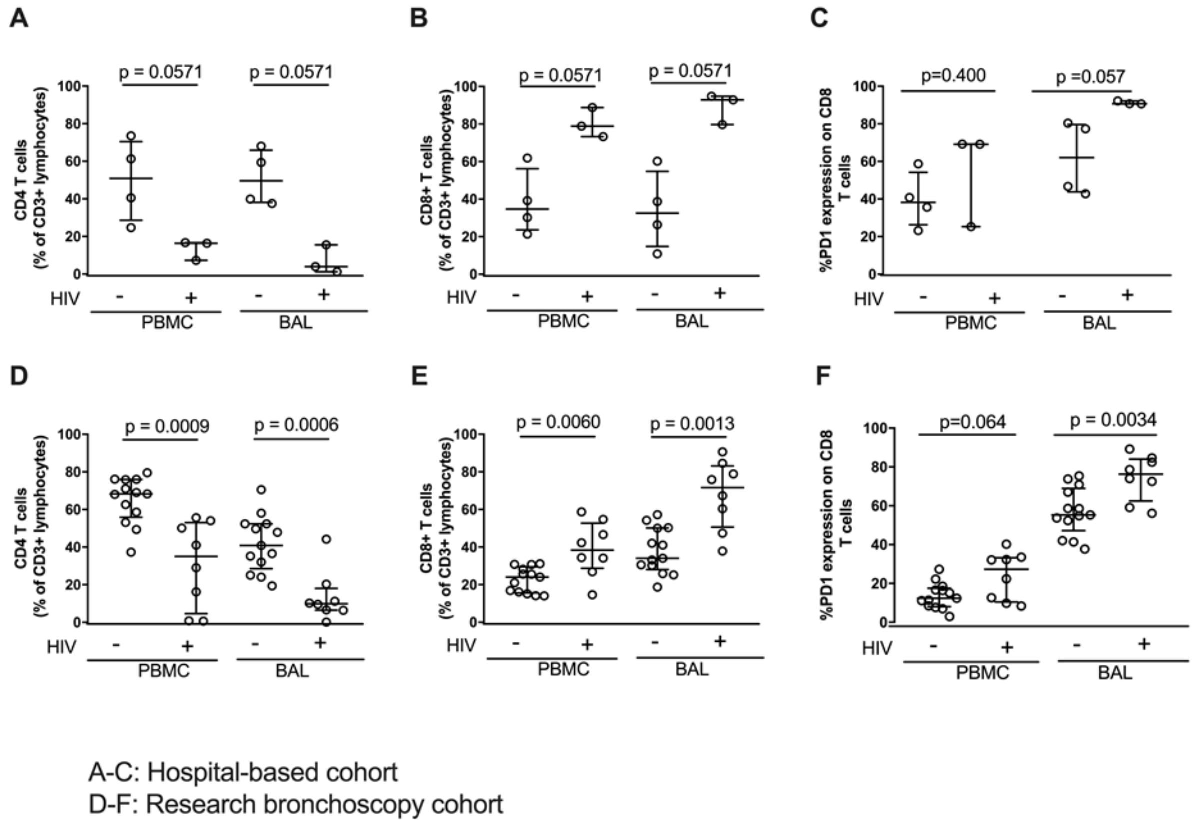

Supplementary Figure 1: Comparison of the two bronchoscopy cohort HIV infection effect in T cell distribution and phenotype. A-C: Shows the hospital-based bronchoscopy cohort CD4 T cells, CD8 T cells distribution and CD8 T cells PD-1 expression in the peripheral blood and bronchoalveolar compartment in HIV-negative and HIV-positive people. D-F: Shows the Research bronchoscopy cohort CD4 T cells, CD8 T cells distribution and CD8 T cells PD-1 expression in the peripheral blood and bronchoalveolar compartment in HIV-negative and HIV-positive people.

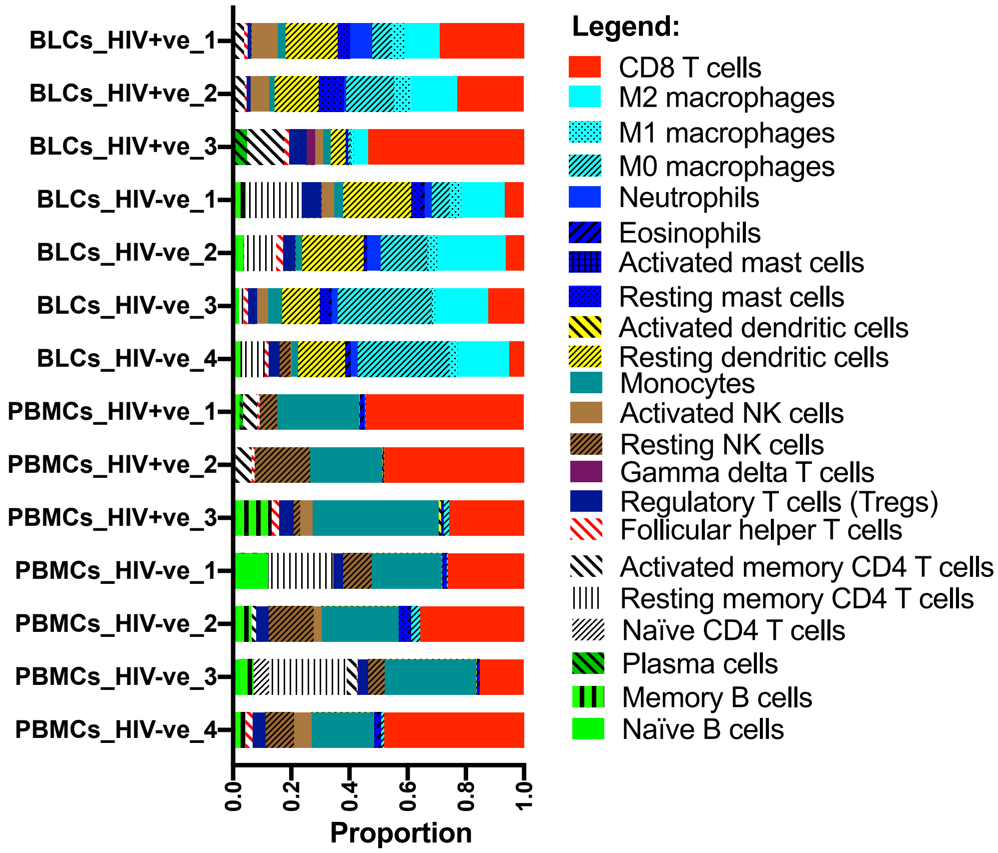

Supplementary Figure 2: Deconvolution analyses of bulk RNA-seq samples to estimate the proportions of 22 leukocyte populations.

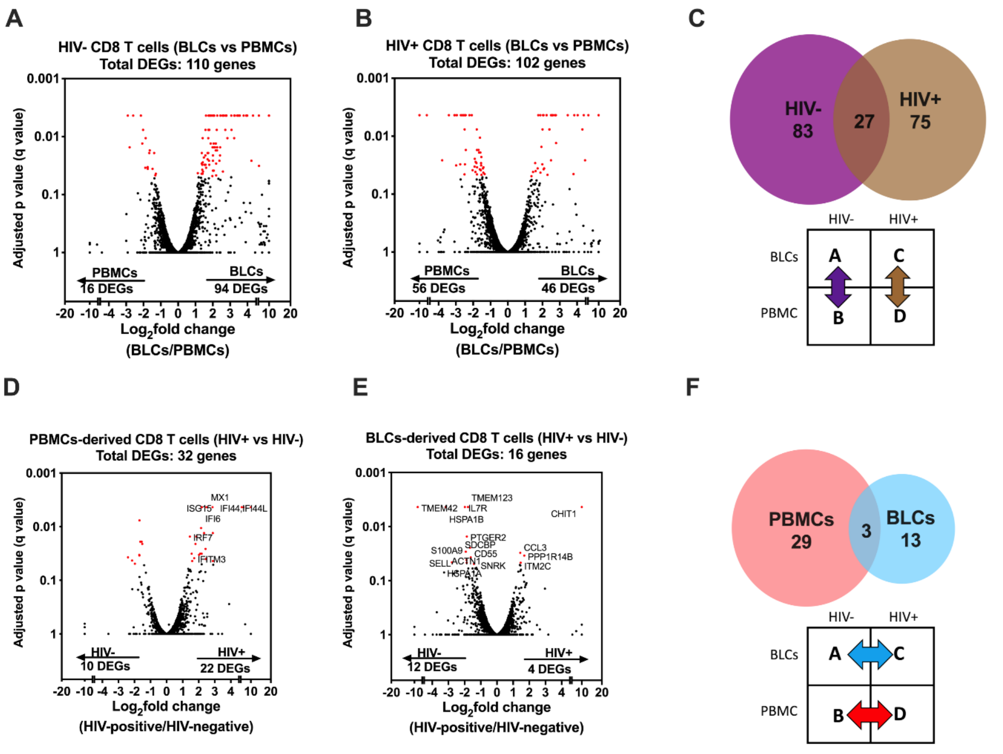

Supplementary Figure 3: Comparisons in transcriptomic profiles between BLCs-derived CD8 T cells and PBMCs-derived CD8 T cells in HIV-infected and HIV-uninfected participants. A) Differentially expressed genes between BLCs-derived CD8 T cells and PBMCs-derived CD8 T cells in the HIV-negative group (n=4). B) Differentially expressed genes between BLCs-derived CD8 T cells and PBMCs-derived CD8 T cells in the HIV-positive group (n=3). C) Numbers of differentially expressed genes between BLCs-derived CD8 T cells and PBMCs-derived CD8 T cells in both HIV-positive (n=3) and HIV-negative groups (n=4). D) Differentially expressed genes between HIV-negative (n=4) and HIV-positive (n=3) groups in PBMCs-derived CD8 T cells. E) Differentially expressed genes between HIV-negative and HIV-positive groups in BLCs-derived CD8 T cells. F) Numbers of differentially expressed genes between HIV-negative and HIV-positive groups in both PBMCs-derived CD8 T cells and BLCs-derived CD8 T cells.

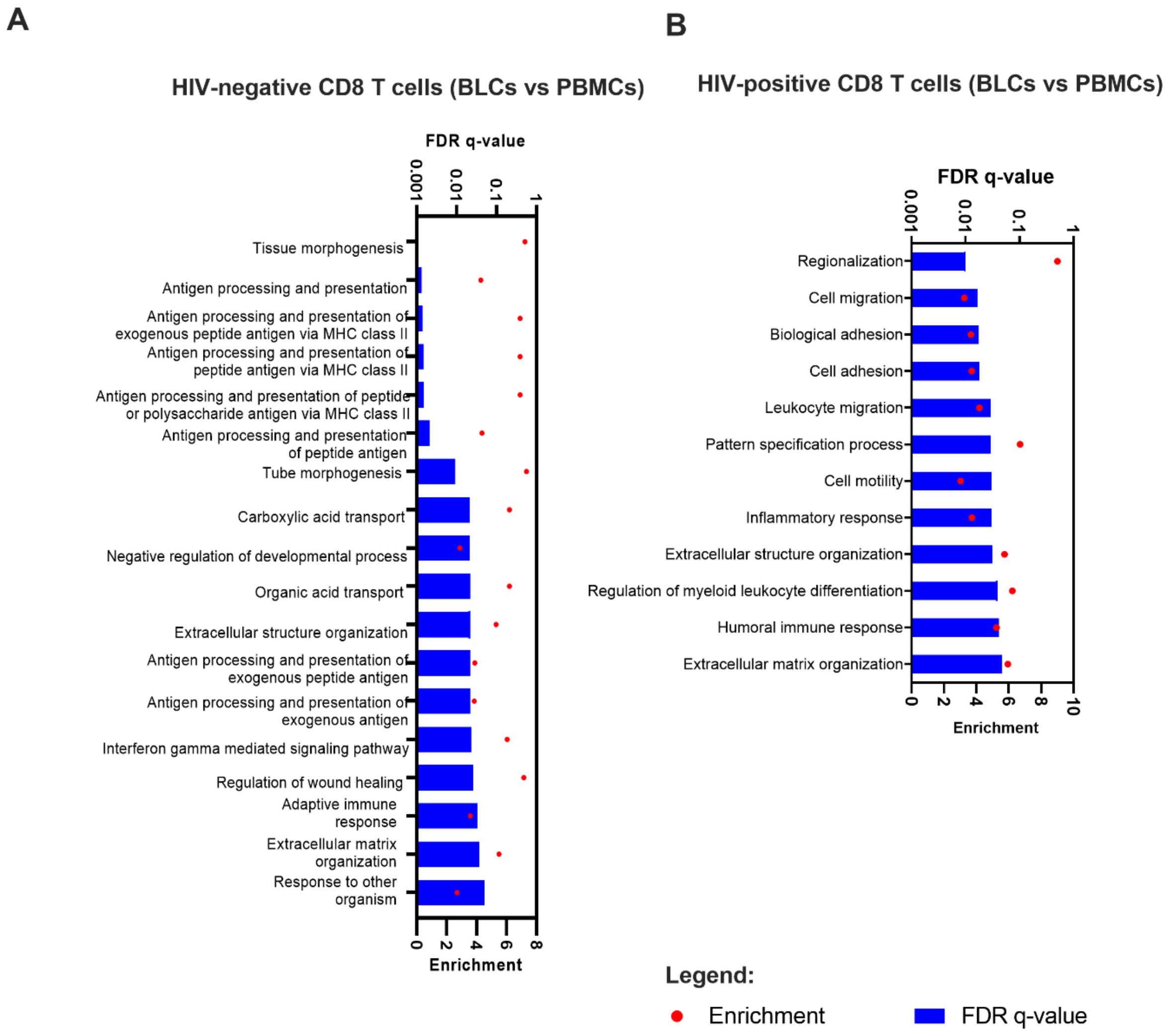

Supplementary Figure 4: Gene ontology analyses to identify compartment-specific functional enrichments in CD8 T cells. A) Enriched gene ontology (GO) terms in comparisons between BLCs-derived CD8 T cells and PBMCs-derived CD8 T cells in HIV-negative individuals. B) Enriched gene ontology (GO) terms in comparisons between BLCs-derived CD8 T cells and PBMCs-derived CD8 T cells in HIV-positive individuals.

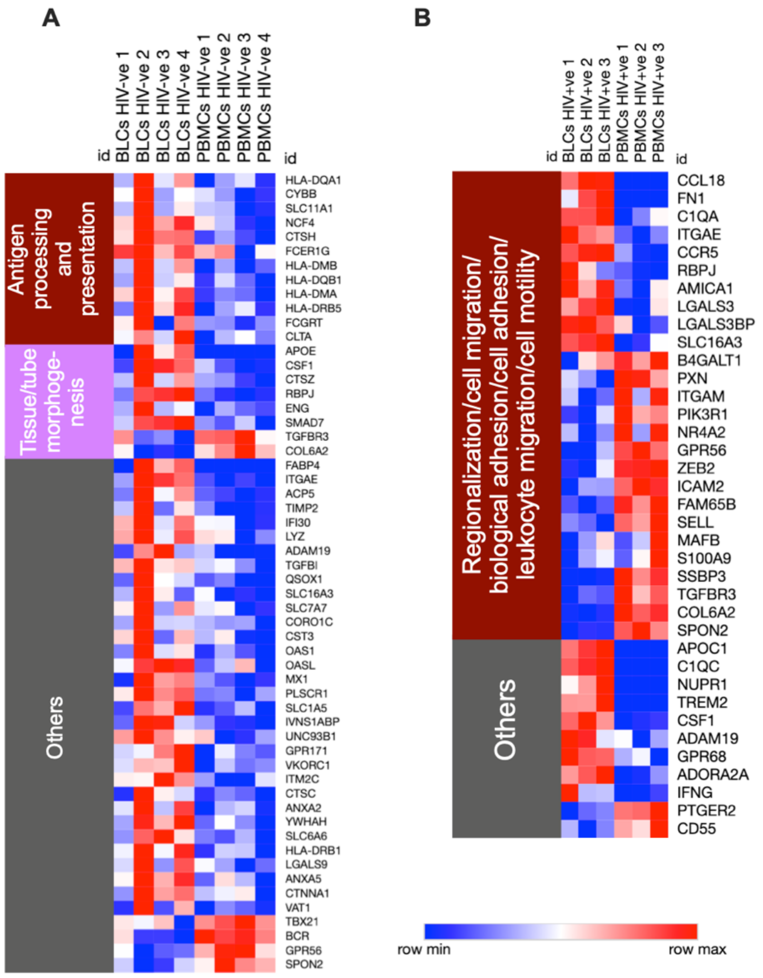

Supplementary Figure 5: Heat maps showing expression levels of the genes that contribute to the enriched GO terms in comparisons between PBMCs-derived CD8 T cells and BLCs-derived CD8 T cells. A) HIV-negative individuals. B) HIV-positive individuals. Natural logarithms of (Expression level +1) were used.
