# Supplementary figures and images for: Contrasting Inflammatory Signatures in Peripheral Blood and Bronchoalveolar Cells Reveal Compartment-Specific Effects of HIV Infection
Source: Front Immunol. 2020 May 19;11:864. doi: 10.3389/fimmu.2020.00864 (PMC7248324; doi:10.3389/fimmu.2020.00864)

## Slide 1
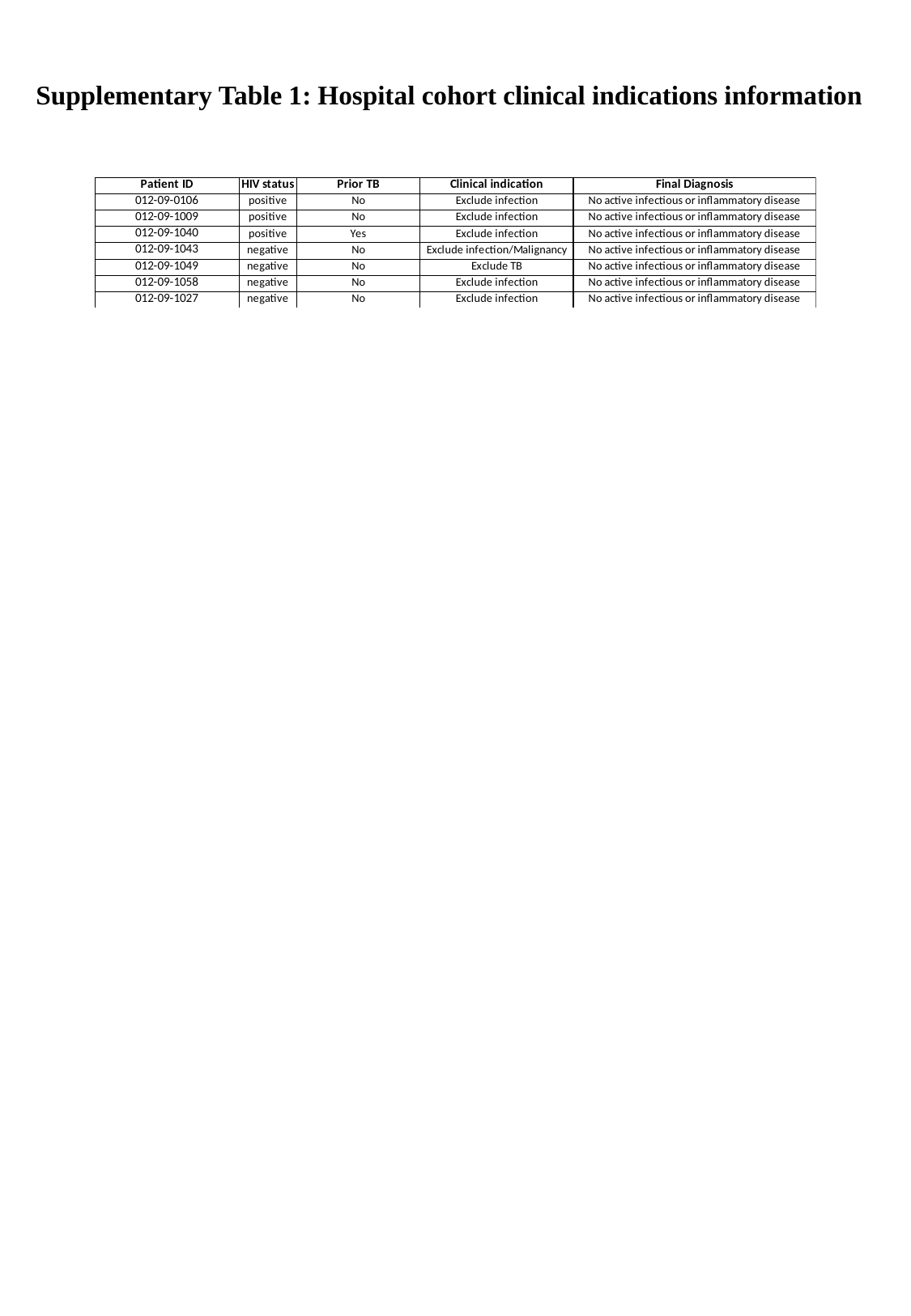

Supplementary Table 1: Hospital cohort clinical indications information

Supplement: Supplementary file 2 [file Presentation_1.PPT]
