# Supplementary material for: Contrasting Inflammatory Signatures in Peripheral Blood and Bronchoalveolar Cells Reveal Compartment-Specific Effects of HIV Infection
Source: Front Immunol. 2020 May 19;11:864. doi: 10.3389/fimmu.2020.00864 (PMC7248324; doi:10.3389/fimmu.2020.00864)
Supplement: Supplementary file 3 [file Presentation_2.PPT]

## Slide 1
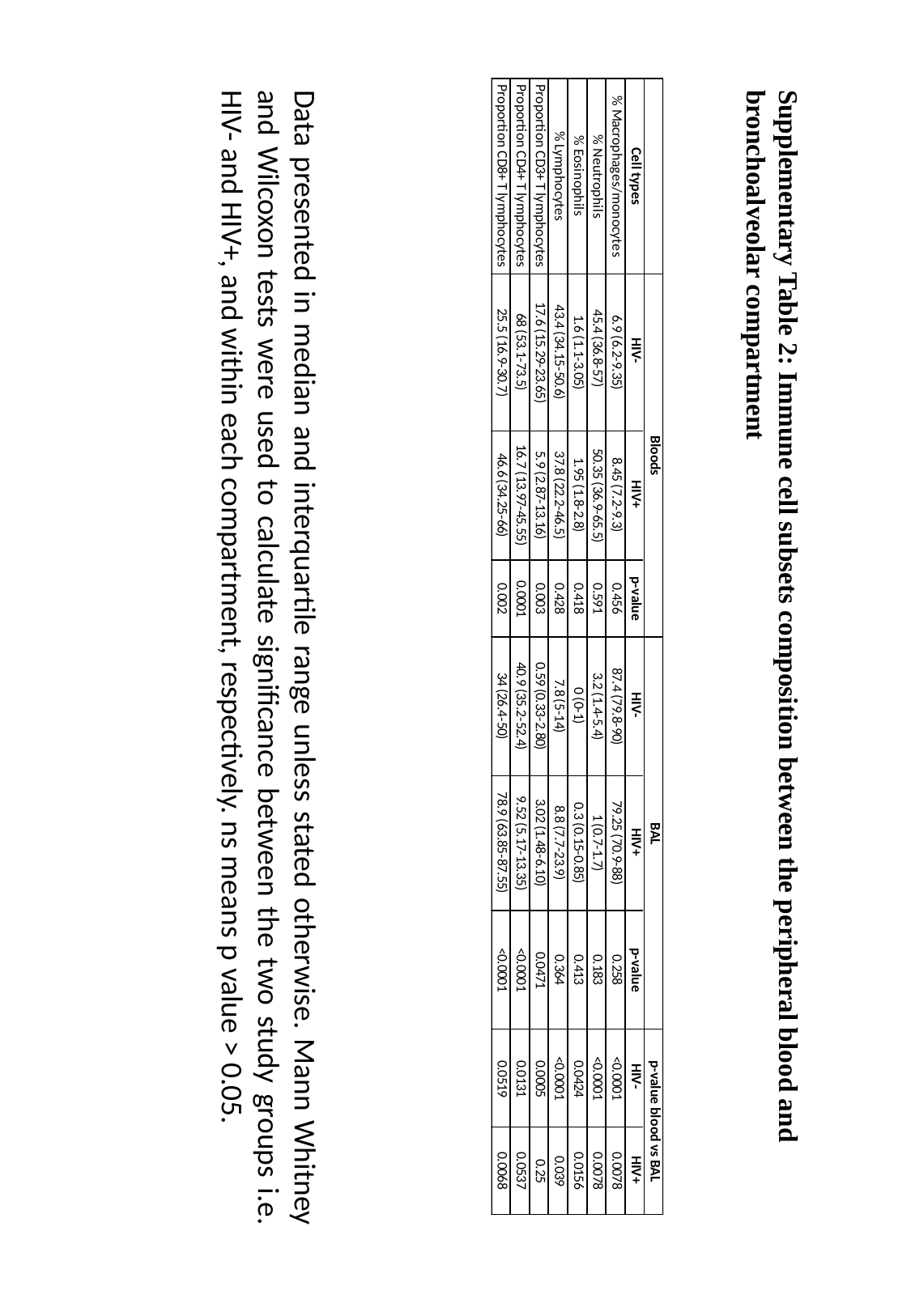

Data presented in median and interquartile range unless stated otherwise. Mann Whitney and Wilcoxon tests were used to calculate significance between the two study groups i.e. HIV- and HIV+, and within each compartment, respectively. ns means p value > 0.05.
Supplementary Table 2: Immune cell subsets composition between the peripheral blood and bronchoalveolar compartment
